# The Clinical Pathological Characteristics and Prognostic Relevance of Homologous Recombination Repair Gene Mutations in Ovarian Cancer Patients: A Prospective Cohort Study

**DOI:** 10.1155/ogi/5578247

**Published:** 2025-03-24

**Authors:** Shitong Zhan, Feng Chen, Lijuan Huang, Lin Chen, Haoyi Jia, Shaofei Ma, Min Tang, Chongzhi Zhou, Yanmin Chen, Ye Yang

**Affiliations:** ^1^Obstetrics and Gynecology Department, Shanghai General Hospital, Shanghai Jiao Tong University School of Medicine, 85 Wujin Road, Hongkou, Shanghai 200080, China; ^2^Pathology Department, Shanghai General Hospital, Shanghai Jiao Tong University School of Medicine, 85 Wujin Road, Hongkou, Shanghai 200080, China; ^3^Surgery Department, Shanghai General Hospital, Shanghai Jiao Tong University School of Medicine, 85 Wujin Road, Hongkou, Shanghai 200080, China; ^4^Educational Department, Shanghai General Hospital, Shanghai Jiao Tong University School of Medicine, 85 Wujin Road, Hongkou, Shanghai 200080, China

**Keywords:** BRCA, homologous recombination(HRR), ovarian cancer, PARPi, prognosis

## Abstract

**Backgrouds:** Whether homologous recombination repair (HRR) mutation has a differential effect on the prognosis has not been confirmed by current studies. The purpose of this study was to explore the clinical importance, prognostic value, and frequency of pathogenic changes in HRR genes in patients with ovarian cancer (OC).

**Methods:** We analyze information including HRR mutation and clinical prognosis of OC patients both in our cohort and in the TCGA-OV database. Blood and/or tumor samples from 98 women admitted to Shanghai General Hospital between January 2021 and May 2024, and DNA sequencing was performed on these samples for all patients included in this retrospective study. Testing was performed for HRR mutations, including germline BRCA1/2 mutations, and defects in HRR were defined as detrimental mutations within relevant genes. Comprehensive medical records were gathered for all patients, with a follow-up period recorded for 74 of them.

**Results:** HRR pathway genes, including BRCA1/2, CDK12, RAD54L, RAD51, ATM, MRE11, and BRIP2, are highly expressed in FIGO Stages I-II OCs among 482 patients in the TCGA-OV database, and 95.06% samples presented mutations. The alignment diagram analyzed by logistic and Cox regression was derived to investigate HRR genes on overall survival (OS < 763 days) of OC patients. A total of 98 patients were enrolled in our study, with 70 harboring HRR mutations (HRRmt) and 28 having the HRR wild-type (HRRwt). The predominant pathological type across all four patient groups was high-grade serous adenocarcinoma, with similar prevalence in HRRmt (84.30%) versus HRRwt (75%, *p*=0.360) and BRCAmt (94.20%) versus BRCAwt (74.60%, *p*=0.151) groups. Survival prediction data were collected from 74 patients, and the HRRmt group (*n* = 50) exhibited a numerically longer PFS compared to the HRRwt group (*n* = 24), with 23 months versus 17 months, respectively. A significant disparity was noted in the percentage of patients administered PARPi medication between the HRRmt and HRRwt groups (58.00% vs. 20.20%; *p*=0.003). Patients in both the HRRmt group (*p*=0.049) and the BRCAwt group (*p*=0.046) receiving PARPi treatment have extended PFS. Significant differences were identified between HRRmt and HRRwt groups in the size of the initial debulking surgery achieving R0 status (*p*=0.005), low CA125 levels (< 1000 U/mL) at diagnosis (*p*=0.015), and the use of PARP inhibitors (PARPi) (*p*=0.024) and antiangiogenic drugs (*p* < 0.001). For patients with HRR mutations, the use of PARPi significantly impacted PFS (*p*=0.049), and achieving R0 status (*p*=0.005) significantly influenced PFS.

**Conclusions:** This study indicates that mutations in the HRR gene possess significant potential as a prognostic marker in OC. Our aim was to comprehensively explore how HRR gene mutations, including but not limited to BRCA, might influence the clinical course and survival of patients, shedding light on potential new avenues for personalized treatment strategies.

## 1. Background

Ovarian cancer (OC) stands as the most lethal gynecological malignancy globally [1]. Notably, around 70% of OC patients are diagnosed at an advanced stage, primarily due to the absence of early symptoms [2]. Despite undergoing standard treatments such as cytoreductive surgery and platinum-based chemotherapy, almost 70% of patients with late-stage OC experience recurrence within 5 years [3], with 5 year survival rate following recurrence below 10% [4] due to nonspecific clinical manifestations, inadequate diagnosis, and the absence of efficient screening methods for early disease detection.

Homologous recombination repair (HRR) is essential for repairing DNA double-stranded breaks. Mutations in HRR-related genes, such as BRCA1/2, RAD51, and MRE11, disrupt this process, resulting in impaired HRR function known as homologous recombination deficiency (HRD), which signifies a malfunction in the HRR pathway. HRD should not be solely defined by the response to any one therapy, given that many studies have shown that HRD has both a positive prognostic value in ovarian and other cancers and predictive value for PARPi and platinum therapy. In addition, testing capabilities may evolve to better assess HR status with a functional assay. The implementation of poly (ADP-Ribose) polymerase (PARP) in the treatment of high-grade serous ovarian tumors characterized by HRD underscores the therapeutic importance of this strategy. Tailoring the choice of concurrent medication to complement PARPi based on individual biomarker status can enhance the effectiveness of OC treatment [5]. While the 2025 National Comprehensive Cancer Network (NCCN) guidelines for OC underscore that the use of PARP inhibitors as adjuvant or maintenance therapy for OC has been elevated to a first-tier recommendation, regardless of the patient's HRD status. This updated approach has demonstrated the ability to induce complete clinical remission in 80% of OC patients [6]. Specifically, Niraparib has exhibited significant improvements in progression-free survival (PFS) and can dramatically decrease the risk of disease progression or death by up to 60% in OC patients harboring BRCA mutations [7].

In addition, with the rapid development of genomics technology, a comprehensive analysis of genomic variations in OC patients has become possible. The landscape of genetic alterations in cancer is vast and complex, with many patients harboring mutations in genes other than BRCA. The NCCN guidelines advise testing for 15 HRR-related genes, including BRCA1, BRCA2, and TP53. In previous studies, it has been suggested that mutations in the BRCA genes may hold a significant association with the patient prognosis. Beyond harmful mutations in genes such as BRCA1/2, it is recognized that HR deficiency imprints OC with specific patterns of base substitutions and structural chromosomal variations. Genes involved in the HRR pathway were widely distributed and intersect with other pathways, but the impact of HRR genes associated with the risk of OC is unclear. Also, the efficacy and safety of different PARP inhibitor-based combinations in patients with HRR mutations have not been evaluated in OC. We conducted a retrospective study along with the TCGA-OV database and aim to analyze in detail the variations of HRR genes such as BRCA1, BRCA2, and TP53 in OC patients in order to explore the relationships between these gene variations and patients' clinical characteristics and explore the prognostic value of pathogenic HRR gene mutations in OC patients.

## 2. Materials and Methods

### 2.1. HRR Gene Mutations in the TCGA-OV Database

We examined the correlation between overall survival (OS) and tumor mutational burden (TMB) among the 482 patients as well as analyzed the types and the proportions with HRR gene mutations using the TCGA-OV database.

### 2.2. Study Design and Treatments

This study retrospectively collected data from the inpatient electronic medical record system (Nanjing Haitai Medical Information System) spanning from January 1, 2021, to May 31, 2024. The data pertain to OC patients treated at the Shanghai General Hospital affiliated to Shanghai Jiao Tong University School of Medicine. The Institutional Review Board (IRB) of the hospital approved this study (2024022). This study is a scientific research project with a cohort study design, combining a retrospective and prospective research approach. Throughout the study, our patient follow-up and treatment were conducted in strict accordance with the NCCN guidelines to ensure the scientificity and accuracy of the study. To ensure the legality and ethics of the study, all the patients participating in this study voluntarily signed the informed consent form on the basis of full knowledge of the study content.

**Figure 1:**
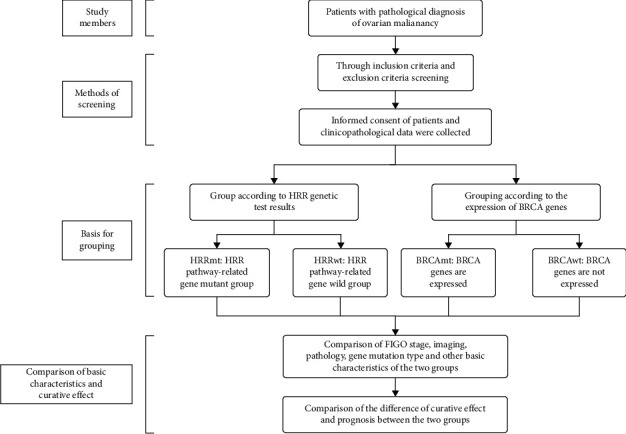
CONSORT diagram of the trial. The flow diagram depicts the disposition of patients throughout the phases of the study, including enrollment, allocation, and follow-up. Patients included in the intention to treat the analysis were evaluated for DFS and PFS.

A detailed flowchart of the study is presented in Figure 1. The collected detailed information mainly includes the following: (1) basic personal information; (2) 2014 International Federation of Gynecology and Obstetrics (FIGO) staging at the time of initial diagnosis; (3) histological types and grade; (4) disease details at the initial diagnosis or recurrence, including tumor locations, maximum diameters (cm) of primary and metastatic lesions (following RECIST criteria), and CA125 progressive elevation [8]; (5) details of tumor cell debulking during surgeries, categorized as R0 (complete debulking), R1 (macroscopic residual disease visible after complete resection, with residual lesion ≤ 1 cm), and R2 (macroscopic evidence of the tumor at the resection margin); (6) carbohydrate antigen 125 (CA125) levels at the initial diagnosis and at the conclusion of each chemotherapy cycle. In this study, four to six chemotherapy sessions constituted one chemotherapy cycle; (7) immunohistochemistry (IHC) results, including markers such as ER, PR, P16, P53, P63, PAX8, CK7, and CEA; (8) details of chemotherapy cycles and platinum-based regimens, considering four to six sessions as one cycle; (9) information on whether PARP inhibitors and antiangiogenic drugs such as bevacizumab were used as maintenance therapy (Figure 1).

### 2.3. Subject Eligibility Criteria

Inclusion criteria include individuals diagnosed with primary or recurrent OC and availability of comprehensive and clinically traceable medical records. (1) Pathological confirmation: patients with OC (either newly diagnosed or recurrent), (2) patients with complete medical records and available for follow-up on clinical outcomes, and (3) patients who have completed genetic testing at our hospital and signed the informed consent form. Exclusion criteria include individuals who are no longer traceable for follow-up.

### 2.4. Germline/Somatic BRCA (g/sBRCA) Testing and HRR Deficiency Testing

The study participants were categorized based on HRR gene variations in their tumor tissue. Annotations followed the ACMG and AMP 2015 guidelines on sequence variant interpretation which encompassed various genes including BRCA1, BRCA2, RAD51B, RAD51C, RAD51D, RAD54L, ATM, ATR, AR, ERBB2, BRIP1, CDK12, KRAS, PTEN, STK11, TP53, BRAF, NRAS, PIK3CA, PALB2, TSC2, FANCA, MRE11, BARD1, CDH1, CHEK, ESR1, FANCL, HDAC2, HOXB13, NBN, and PPP2R2A. Two primary grouping methods were utilized to explore clinicopathological associations and patient outcomes: (1) HRR mutation status: Participants were divided into two groups—those with HRR pathway–related gene mutations (HRRmt) and those with the wild-type HRR gene (HRRwt). (2) BRCA mutation status: Similarly, patients were classified based on whether they had BRCA gene mutations (BRCAmt) or the wild-type BRCA gene (BRCAwt).

### 2.5. Follow-Up

The follow-up schedule based on the NCCN guidelines process involves reviewing medical records, outpatient data, and conducting telephone interviews. The scope of the follow-up encompasses the following: detailed medical history inquiries, transvaginal color doppler ultrasound assessments, monitoring of tumor markers (specifically, CA125 and HE4 levels), and imaging tests such as pelvic and abdominal high-resolution pelvic magnetic resonance imaging (MRI), whole-body 18-fluorine-fluorodeoxyglucose positron emission tomography/computed tomography (18F-FDG PET/CT), and enhanced abdominal CT. PFS is measured from the date of the first diagnostic surgery until imaging or serum markers exceed normal thresholds (CA125 > 30.2 u/mL, HE4 > 140 u/mL). Disease-free survival (DFS) spans from the commencement of the patient's initial treatment until the final follow-up, accounting for instances of death or loss to follow-up.

### 2.6. Statistical Analysis

Statistical analysis was conducted using SPSS Statistics 30.0 and GraphPad Prism 10.0.0. Descriptive statistical methods summarized patients' general information. Independent sample *t*-tests or Mann–Whitney *U* tests were utilized for comparing continuous variables between HRRmt and HRRwt groups. Pearson chi-square tests were conducted on association between different categorical variables and patient relapse. To ensure the accuracy of the results, we also employed Yates-corrected chi-square tests and Fisher's exact tests for supporting the analysis and correction upon small sample size. Cox regression analysis was performed for PFS and DFS, hazard ratio (HR), 95% confidence interval (CI), PFS, and DFS were calculated. The Kaplan–Meier method was applied to analyze survival in different groups. TMB for OC was calculated using the “forestplot” *R* package. The expression of HRR-related genes, mutation landscapes, and survival curves were visualized using the “heatmap,” “survival,” “maftools,” and “DynNom” *R* packages. Nomograms were analyzed by logistics regression and Cox regression. The binary logistic regression was used to identify differences, with a significance level set at *p* <  0.05.

## 3. Results

### 3.1. HRR Gene Mutations in TCGA Database

**Figure 2:**
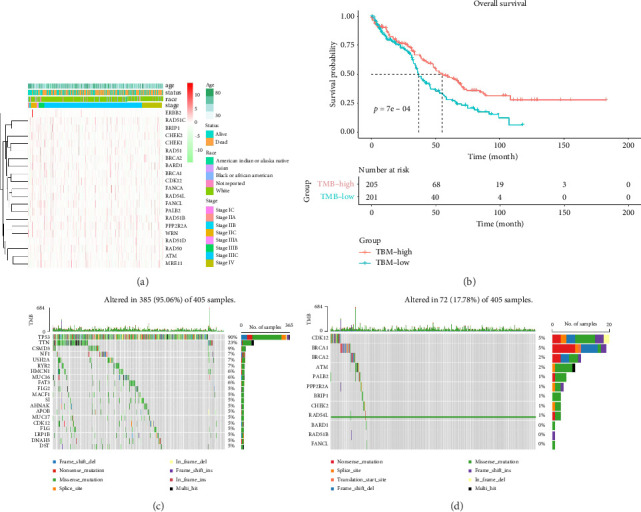
Clustered mutation profile of HRR genes among ovarian cancer patients. (a) The types of mutations of altered patients from the TCGA-OV database. The mutation profile of each patient was presented with respective baseline characteristics including age, status, race, and FIGO stage. (b) A significant disparity was observed in OS between the TMB-low and TMB-high groups (40 months vs. 68 months, *p* < 0.05). (c) The mutation patients (385/405; 95.06%) in the TCGA-OV database. (d) The altered nonmutation patients (72/405; 17.78%) in the TCGA-OV database.

We gathered comprehensive information from 482 patients recorded in the TCGA-OV database. Our results indicated that genes associated with the HRR pathway genes, including BRCA1/2, CDK12, RAD54L, RAD51, ATM, MRE11, and BRIP2, are highly expressed in FIGO Stages I-II OCs. Notably, PPP2R2A expression remained stable throughout all stages. Furthermore, RAD51D exhibited notably elevated expression in Stage IV (Figure 2(a)). TMB is a measure of the density of asymmetric mutations within protein-coding regions, emerged as a promising immunotherapy target. To explore this further, we examined the correlation between OS and TMB among the 482 patients. By stratifying the data based on the median TMB value, we uncovered a significant disparity in OS between the TMB-low and TMB-high groups (40 months vs. 68 months, *p* < 0.05) (Figure 2(b)). In addition, we analyzed the types of mutations and the proportions of patients with common gene mutations using the TCGA-OV database. Our results indicated that mutations were present in 385 out of 405 samples (95.06%) (Figure 2(c)).

Notably, among 405 out of 482 samples in the TCGA-OV database, 72 instances (17.78%) exhibited alterations in the HRR gene. Approximately 90% of these mutations were attributed to TP53 alterations, with missense mutations accounting for roughly 55% of these TP53 mutations. As for other genes, their mutation rates varied between 5% and 9% (Figure 2(d)). Specifically, nonsense mutations, frameshift deletions, and frameshift insertions together accounted for less than 30% of all mutations, with CDK12 mutations contributing 5%. Missense mutations and frameshift insertions were less frequent than BRCA1 mutations, which made up 5% of the total. Approximately 40% of all mutations were identified as nonsense mutations, and 30% as frameshift deletions.

### 3.2. The Impact of HRR Genes on Prognosis in TCGA

**Figure 3:**
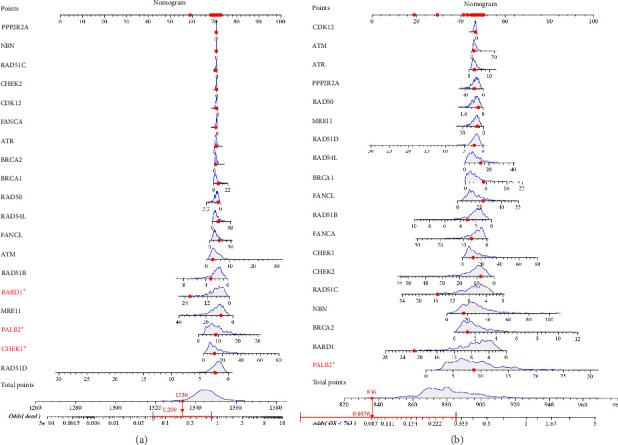
Nomogram of the HRR gene on the TCGA-OV database. (a) Results of logistics analysis; genes such as RAD51D, CHEK1, and PALB2 emerge as significant predictors of death probability. Higher expression of these genes might indicate a higher likelihood of death. (b) Results of the Cox regression analysis. Genes such as PALB2, BARD1, and BRCA2 are a key in predicting overall survival. Higher expression of PALB2 and lower expression of BARD1 and BRCA2 might be associated with a better prognosis.

To investigate whether various HRR genes consistently influence the survival status of OC patients, we generated the alignment diagram analyzed by logistic and Cox regressions. These analyses utilized gene expression data and clinical information from 429 patients in the TCGA OC database. For the alignment diagram derived from the logistic regression analysis, we assessed the impact of each HRR gene on survival status. The genes that contributed in descending order were RAD51D, CHEK1, and ATM. Furthermore, the scores of RAD51B (point = 68), BARD1 (point = 60), MRE11 (point = 72), and RAD51D (point = 70) were negatively correlated with the probability of death, while the scores of CHEK1 (point = 70), PALB2 (point = 70), and ATM (point = 70) were positively correlated with the probability of death. The total point of all HRR genes is 1330, corresponding to odds of dead being 0.209 (Figure 3(a)). For the alignment diagram derived from the Cox regression analysis, we evaluated the effect of each HRR gene on OS (OS < 763 days). The genes contributing in descending order were PALB2, BARD1, and BRCA2. In addition, scores such as BARD1 (point = 19), RAD51D (point = 46), and RAD51C (point = 29) were negatively associated with the probability of OS < 763 days, whereas scores such as PALB2 (point = 48), ATM (point = 46), and BRCA2 (point = 44) were positively associated with the probability of OS < 763 days. The total point of all HRR genes is 836, corresponding to odds of OS < 763 days being 0.0876 (Figure 3(b)). Therefore, we suspected upregulation of RAD51 family members and downregulation of PALB2, the expression of ATM and BRCA2 might predicate a well prognosis.

### 3.3. HRR Gene Mutations in Cohort

**Figure 4:**
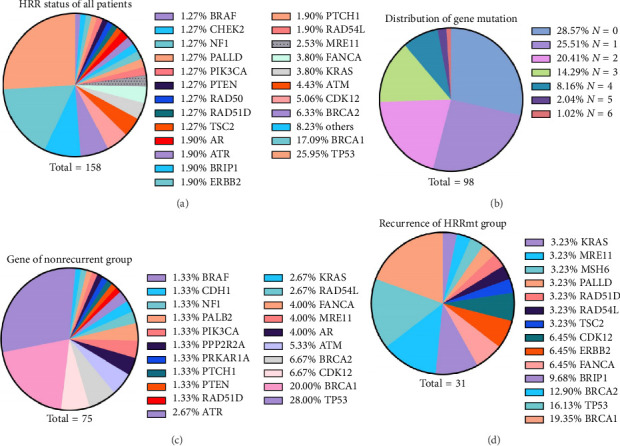
Distribution on HRR gene mutations. (a) HRR gene mutations of all patients; the highest proportion of BRCA mutations was followed by TP 53; most of the gene mutations are single mutations (25.51%). (b) Numerical distribution of genetic mutations in all patients. (c) The gene distribution of nonrecurrence patients; the highest proportion of BRCA mutations was followed by TP 53. (d) The gene distribution of recurrence patients in the HRRmt group.

From January 2021 to May 2024, a total of 98 patients participated in the study. We detected 158 gene mutations among all the patients, results were available with respect to both germline or somatic BRCA (g/sBRCA) testing and HRR deficiency testing. A notable 71.43% (70 out of 98) were found to have at least one deleterious mutation in their HRR genes. Thirty-six distinct pathogenic or potentially pathogenic HRR gene mutations were identified. Notably, TP53 stood out as the gene with the highest mutation frequency, affecting 41 out of 158 gene mutations (25.95%). This included 12 instances of single-gene mutations and 29 cases where it was mutated alongside other genes. Following closely, BRCA 1/2 mutations were observed in 37 out of 158 gene mutations (23.42%). Among the other HRR genes, CDK12, ATM, KRAS, and FANCA accounted for respective mutation rates of 8/158 (5.06%), 7/158 (4.43%), 6/158 (3.80%), and 6/158 (3.80%). In addition, 53/158 (33.54%) mutations were attributed to other HRR genes (Figure 4(a). In terms of mutation types, single-gene mutations in HRR genes comprised 25.51% (25/98) of the total, while combined mutations involving two HRR genes made up 20.41% (20/98). Furthermore, mutations impacting three or more HRR genes were observed in 25.51% (25/98) of the patients (Figure 4(b)). There were 15 recurrent events in the HRRmt group and nine in the HRRwt group, respectively. The remaining 50 patients who did not have recurrence, primarily had gene mutations in TP53 (28.00%), followed by BRCA (26.67%), CDK12 (6.67%), and ATM (5.33%) (Figure 4(c)). When comparing the genetic composition of recurrent patients (*N* = 24) to that of the nonrecurrent group (*N* = 50), we observed that in the recurrent group, BRCA mutations were most common (32.25%), followed by TP53 (16.13%) and RAD51D/54L (6.46%) (Figure 4(d)).

Within the HRRmt group, pathogenic variants were categorized as follows: 5-class (13.29% or 21/158), 4-class (39.87% or 63/158), and 1–3 classes (46.83% or 74/158). It is noteworthy that 53.17% of the HRRmt group exhibited pathogenic or potentially pathogenic HRR mutations. Regarding BRCA mutations, a significant proportion of 70.00% was classified as four to five class variants. Similarly, among TP53 mutations, a predominant 88.46% fell into the four to five class variant category. Across all mutation status observed in FIGO late-stage patients, who recurred a total of 32 mutated genes, were identified. These mutations comprised 46.67% missense mutations, 16.67% frameshift mutations, 10.00% nonsense mutations, and 10.00% synonymous mutations (Supporting Table 1).

We conducted an exploration of the clinicopathological correlations and patient outcomes utilizing two distinct classification methods. Based on whether the HRR gene is mutated, we divided the patients into two groups: the HRR mt group (*n* = 70) and the HRRwt group (*n* = 28). Similarly, based on whether the BRCA gene is mutated, the patients were classified into the BRCA mt group (*n* = 63) and the BRCAwt group (*n* = 35). The predominant pathological type observed across all four patient groups was high-grade serous adenocarcinoma (HRRmt vs. HRRwt, 84.30% vs. 75%, *p*=0.360). Similarly, the BRCAmt group showed a prevalence of 94.20% versus 74.60% in the BRCAwt group (*p*=0.151). A striking finding emerged in the form of a significant difference in P16 IHC expression, with 30 out of 70 patients in the HRRmt group demonstrating positive, when comparing recurrence between the HRRmt and HRRwt groups (*p*=0.004). Furthermore, no statistically significant differences of patients' recurrence were observed between the two groups with respect to the number of chemotherapy cycles administered, the use of platinum drugs, the incorporation of bevacizumab, serum CA-125 levels, FIGO stage, the maximum primary tumor diameter at the time of diagnosis, or the achievement of R0 resection status (Table 1).

### 3.4. The Impact of HRR Genes on Prognosis in Cohort

**Figure 5:**
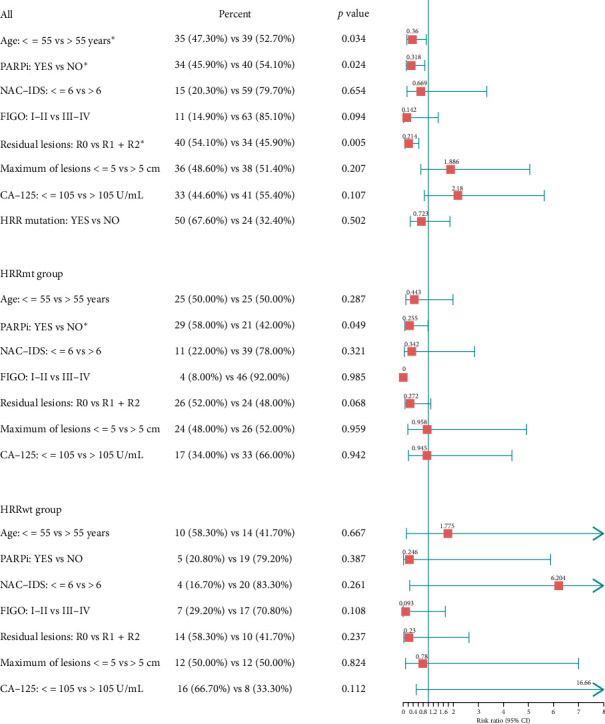
Prespecified subgroup analysis of PFS according to the HRR mutation status. HRs for the effective factors on PFS according to patient characteristics are shown. Red squares denote HR and whiskers indicate 95% two-sided CI. There were significant *p* values for PARPi application (*p*=0.024) and surgical reduction up to R0 (*p*=0.005).

**Figure 6:**
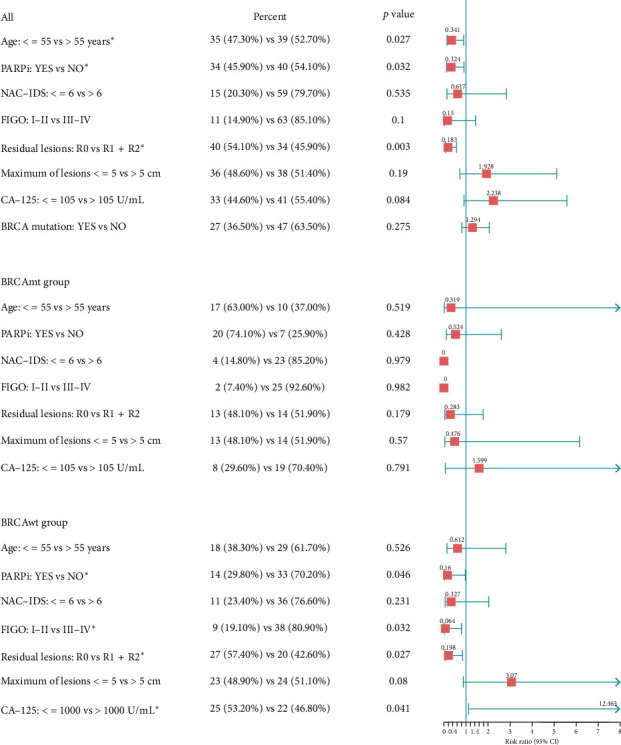
Prespecified subgroup analysis of PFS according to the BRCA mutation status. There were significant *p* values for PARPi application (*p*=0.032) and surgical reduction up to R0 (*p*=0.003).

In addition, we compared PFS and DFS between the HRRmt and HRRwt groups to explore the potential association between HRR gene mutations and patient prognosis. Cox regression multivariate analyses were performed on the 74 patients with survival follow-up data to determine the most significant predictors of extended PFS and DFS. The HRRmt group exhibited a numerically longer PFS compared to the HRRwt group, with 23 months versus 17 months, respectively (HR = 0.962; 95% CI, 0.410–2.255; *p*=0.929), though it was not statistically significant. Likewise, the DFS was also longer in the HRRmt group (HR = 1.263; 95% CI, 0.529–3.035; *p*=0.601). The initial analysis concentrated on identifying the most influential factors predicting prolonged PFS. A significant disparity was noted in the percentage of patients administered PARPi medication between the HRRmt and HRRwt groups (58.00% vs. 20.20%; *p*=0.003), since 54% (27/50) of patients in the HRRmt group had concomitant BRCA mutations and among them, 11 received PARPi treatment. Patients carrying germline or somatic mutations in BRCA1/2 generally achieve better therapeutic outcomes with PARP inhibitor therapy. Across all patients in the study, patients who receive PARPi treatment have extended PFS (HR, 0.318; 95% CI, 0.118–0.861; *p*=0.024). This finding was consistent within both the HRRmt group (HR, 0.255; 95% CI, 0.065–0.992; *p*=0.049) (Figure 5) and the BRCAwt group (HR, 0.160; 95% CI, 0.026–0.968; *p*=0.046) (Figure 6).

In our study, the absence of residual lesions was identified as another predictor of prolonged PFS in all patients (HR, 0.214; 95% CI, 0.073–0.626; *p*=0.005) (Figure 5) and BRCAwt group (HR, 0.198; 95% CI, 0.047–0.831; *p*=0.027) (Figure 6). In addition, 54.05% of patients (40/74) achieved R0 resection of residual lesions during the initial surgery, revealing a statistically significant difference in recurrence rates between the recurrence and nonrecurrence groups (62.00% vs. 37.50%, *p*=0.037). Significantly, upon further DFS analysis, it was revealed that the administration of antiangiogenic drugs, such as bevacizumab, was a notable predictor for all patients in this study, independent of other clinical factors (*p*=0.004). However, there were no statistically significant differences in DFS prognoses based on factors such as residual lesions, preoperative CA-125 levels, FIGO stage, whether or not patients underwent neoadjuvant chemotherapy followed by interval debulking surgery, age at diagnosis, size of the largest primary tumor, or HRR mutation status.

Conversely, for patients without BRCA mutations, other predictors of a prolonged PFS included a low CA-125 level after surgery (HR, 0.198; 95% CI, 0.047–0.831; *p*=0.027) and early FIGO stage (HR, 12.465; 95% CI, 1.11–140.03; *p*=0.032) (Figure 6). Patients across different FIGO stages displayed varying frequencies of HRR mutations: 72.70% (8/11) in Stage IV, 73.10% (38/52) in Stage III, and 36.40% (4/11) in Stages I-II. Among the 23 patients who experienced recurrence in the late stages (FIGO III-IV), recurrence occurred in both the HRRmt and HRRwt groups (15 vs. 8), encompassing six cases with BRCA mutations and five with TP53 mutations. Notably, this study found that recurrence was significantly associated with the presence of TP53 mutations across all patients (*p*=0.031).

For recurrent patients, various strategies were utilized for managing recurrence, including secondary cytoreductive surgery, bevacizumab administration, and intraperitoneal hyperthermic perfusion. Interestingly, no significant difference was observed in PFS among these three approaches (*p*=0.092). Firstly, we discovered that patients with BRCA mutations in the R0 group (indicating no detectable residual tumor postsurgery) had a marginally longer average survival time compared to BRCA wild type-patients (46.055 ± 6.249 months vs. 31.035 ± 2.216 months). It is striking that patients who underwent secondary cytoreductive surgery exhibited a notably longer median DFS compared to patients who received bevacizumab administration or intraperitoneal hyperthermic perfusion (47 months vs. 25 months and 47 months vs. 17 months, *p*=0.003). In summary, our analysis revealed that BRCA mutations might be associated with better survival outcomes in the R0 group, and secondary cytoreductive surgery may provide a survival benefit in terms of DFS for patients with recurrent OC harboring HRR gene mutations.

## 4. Discussion

In this study, we further explored the clinicopathological features and prognostic relevance of HRR gene mutations in OC patients. We have previously reported preliminary results in our previous research, and we have already published a preprint [9]. However, these preliminary findings have not yet undergone peer review and should be interpreted with caution. By expanding the sample size and conducting more in-depth analyses, this study further validates these preliminary findings and provides more comprehensive evidence.

**Figure 7:**
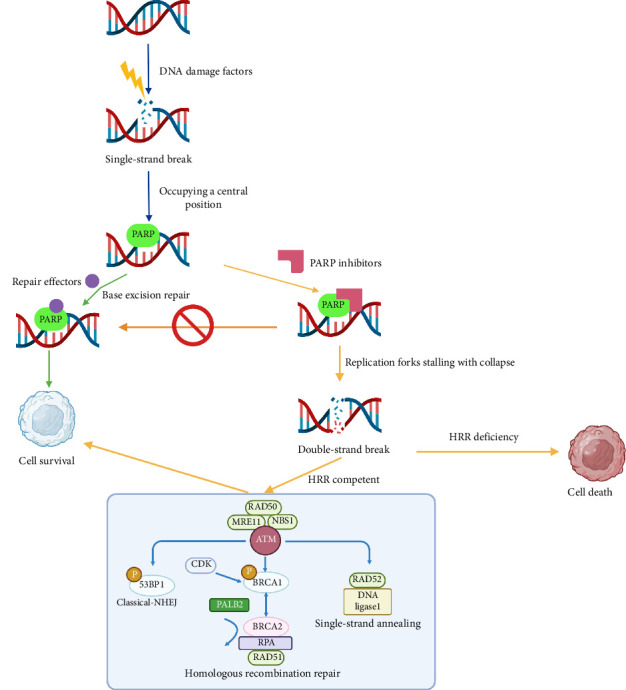
Diagram of the mechanisms of application of PARP inhibitors to induce the death of tumor cell synthesis.

In clinical settings, the BRCA1/2 gene pathogenic mutation and genomic instability score serve as indicators for assessing the HRD status of tumors [10]. The HRD assay plays a crucial role in predicting the efficacy of PARPi in advanced OC treatment [11]. Treating HRD-tumors with PARPi could potentially elevate tumor immunogenicity, suggesting a possible synergistic effect when combined with immunotherapy. When HRD is positive, tumor cells exhibit heightened sensitivity to drugs that induce DNA intersection, such as molybdenum-based chemotherapy. Simultaneously, the use of PARP inhibitors can trigger tumor cell apoptosis, forming the basis for the sensitivity of these cells to treatments such as molybdenum chemotherapy and PARPi (Figure 7). However, the HRD assay has limitations: It requires authorization and may not be accessible due to financial constraints. In addition, it only reflects genomic instability at a specific time and cannot accurately assess changes in HRR functions caused by mutations. While most HRD assays incorporate HRR-associated gene mutations as biomarkers, the distribution and frequency of BRCA and other HRR mutations exhibit notable geographical and ethnic variations, influencing the ethnic HRD rate. Moreover, there is no universally accepted criterion for determining if other mutated gene biomarkers, apart from BRCA1, should be included in the HRD status evaluation.

There are differences in the distribution of BRCA1/2 gene mutations among different races and ethnicities, and the frequency of BRCA1/2 gene mutations is higher in the Jewish population [12]. However, there is a notable lack of comprehensive research on HRR reversion mutations across large pan-cancer cohorts, particularly within the Eastern Asian populace. Most existing studies have either reported sporadic cases or focused on small cohorts primarily from Western populations. A retrospective study conducted on a Chinese pan-cancer patient cohort revealed the presence of reversion mutations in three HRR-associated genes: BRCA1, BRCA2, and PALB2, across breast cancer, pancreatic cancer, OC, and lung cancer [13]. A placebo-controlled Phase III clinical trial [14] revealed that OC patients with defective HRR, due to damaging mutations in any of 16 key genes, exhibited notably longer PFS and OS compared to those without such mutations. In addition to the HRD assay, our study underscores the clinical significance of identifying specifically the HRR gene for prognostic prediction in OC treatment effectiveness. We identified 17.57% pathogenic or potentially pathogenic HRR mutations out of 74 OC patients. Notably, 67.5% harbored various HRR gene mutations and 28.6% accounted for BRCA 1/2 mutations, aligning with 28.5% reported proportion of BRCA 1/2 mutations in Chinese OC patients [15, 16]. Advanced-stage patients were predominantly found in the HRRmt (92%) and HRRwt (70.8%) groups. The most prevalent mutations occurred in TP53 (24.53%), BRCA1 (19.81%), BRCA 2 (8.49%), and CDK12 (6.6%). Germline and somatic mutations affecting HRR genes are relatively commonplace, affecting approximately one-third of ovarian carcinoma patients, with BRCA1 and BRCA2 mutations being the most prevalent [15]. Notably, individuals with inherited BRCA1 and BRCA2 mutations tend to have a longer 5-year survival rate, potentially due to heightened sensitivity to platinum chemotherapy [16]. Our findings align with the GOG-0218 study, which observed that OC patients with defective HRR had significantly extended PFS and OS [14]. Our research dataset also compares favorably with the TCGA data, highlighting the importance of recognizing key genetic predisposition genes for tailored OC treatments.

PARP inhibitors have demonstrated promising therapeutic effects in BRCA-mutated patients, notably extending PFS and OS [17, 18]. Some researchers argue that a patient's sensitivity to platinum medications is a more significant predictor of response, questioning the necessity of HRD testing for forecasting PARPi effectiveness [19]. Currently, there is inadequate evidence supporting the clinical efficacy of PARPi and the predictive value of HRR genes, with some studies even yielding contradictory findings [18, 20]. Notably, the ARIEL3 study demonstrated therapeutic advantages regardless of the mutation status in the exploratory postprogression endpoints [21]. Also, Niraparib has been approved as first-line maintenance therapy for advanced OC patients responding to platinum-based chemotherapy independent of their biomarker status [22]. Data from the Phase IV ORZORA study indicated that Niraparib's clinical benefit was not significant among patients with platinum-sensitive recurrent OC, encompassing those with germline BRCA (gBRCA), systemic BRCA (sBRCA), and HRR-associated (non-BRCA) mutations [23]. The vast majority of HRD assays incorporate HRR-associated gene mutations as biomarkers, the distribution, and frequency of BRCA, and other HRR mutations demonstrate significant geographical and ethnic variations, so the mutational rate contributes to the ethnic HRD rate [24]. Abida et al. advocated that prostate cancer patients with HRR gene defects may benefit significantly from platinum-based chemotherapy and PARP inhibitor treatment, highlighting the therapeutic benefits of targeted therapy for those with DNA repair gene-deficient prostate tumors [25]. Also, Wang et al. revealed that significant mutations leading to HRR deficiency may be a distinct genetic trait in certain primary malignancies among lung cancer patients, representing potential therapeutic targets [26].

Beyond the well-known BRCA1 and BRCA2 genes, mutations in other HRR genes have also been found to be predictive of the patient prognosis and of the effectiveness of PARPi and alternative treatments. TP53 is widely recognized as the gene most commonly mutated in human malignancies, present in at least 50% of human cancers. In this study, we identified 24.53% of patients had TP53 mutations, and among these, 19.2% experienced recurrence, indicating the potential of TP53 missense mutations as a biomarker for monitoring OC clinically [27]. RAD51 and its paralogs, RAD51C and RAD51D, are crucial components of the HRR pathway, forming structures that bind to broken double-stranded DNA [24]. RAD51 is a significant mutated gene in OC, and it is emerging as a promising marker for the functional assessment of HRD in pathology [28, 29]. According to Castroviejo-Bermejo et al., the “nuclear localization” of RAD51 in tumor cells reflects HRR function and assesses resistance to PARPi [30]. In high-grade plasmacytoid ovarian malignancies, RAD51 detection aids in identifying patients' HRD status. The mutation rate of RAD51D ranks third among Chinese OC patients, and patients carrying the RAD51D K91 fs mutation respond well to platinum-based drugs and have a favorable prognosis [31]. CDK12 frequently undergoes biallelic mutation and acts as an oncogene [32]. Its mutation results in loss of kinase activity, decreased BRCA1 expression in OC cells [33], and increased sensitivity to PARPi [34]. Aberrant CDK12 expression or mutation is implicated in cancer initiation, progression, and treatment response. In prostate cancer, inactivating CDK12 mutations correlates with high genomic instability and aggressive clinical features. In addition, CDK12 synergizes with other oncogenes, such as HER2 in breast cancer, to promote tumorigenesis and metastasis. These observations support CDK12 as a therapeutic target in cancer treatment [35]. Cells responding to DNA damage initiate the ATM-CHEK2 cascade response pathway activated by double-stranded breaks to repair broken double-stranded DNA [36]. In the absence of ATM, PARPi is more effective [37]. The MRE11-RAD50-NBS1 (MRN) complex senses DSBs, recruits, and activates ATM at the break site to orchestrate signals in response to DNA damage. Inhibition of MRE11 K673 lactylation boosts the efficacy of chemotherapeutic agents [38].

This study indicated a heightened responsiveness to PARPi among the HRR-mutated group, especially in BRCA-mutated cases, echoing previous findings on the increased sensitivity of BRCA mutation carriers to PARPi [39]. The study further emphasized that the use of PARPi was the primary factor influencing PFS extension in the HRR-mutated cohort. Moreover, patients carrying BRCA1/2 mutations have had more treatment opportunities for both initial and recurrent diseases, including access to novel therapies in clinical trials. Candido-dos-Reis et al. claimed that BRCA1/2 mutations were linked to improved short-term survival, albeit with diminishing benefits over time [40]. In our study, patients with HRR mutations, particularly those harboring BRCA1/2 mutations, exhibited heightened sensitivity to PARPi therapy. Therefore, monitoring the mutation status of HRR genes throughout the disease course could significantly aid in understanding resistance mechanisms and guiding subsequent therapies.

## 5. Practical Applications and Implications for Clinical Practice

The identification of HRR gene mutations as a potential prognostic marker in OC provides a valuable tool for stratifying patients and guiding treatment decisions. The routine screening of HRR gene mutations can aid in early identification of patients with HRD, thus enabling tailored therapeutic approaches. This would facilitate the identification of those patients who are more likely to respond favorably to PARPi and potentially benefit from tailored treatment regimens. Therefore, the integration of HRR gene mutation screening into clinical practice could help identify a subset of OC patients who would derive the greatest benefit from PARPi therapy. Our results underscore the importance of the HRR gene mutation status in guiding PARPi treatment decisions. Patients with confirmed HRR mutations, especially BRCA1/2 mutations, should be prioritized for PARPi therapy. The use of PARPi significantly extended PFS in our HRR-mutated cohort, emphasizing the therapeutic potential of this approach. Clinicians should consider the HRR mutation status when formulating treatment plans, particularly for patients with recurrent or advanced-stage OC.

## 6. Limitation

Given the limited number of OC patients with HRR mutations included in our analysis, the efficacy findings presented in this report should be approached with caution. The functional disparities among various HRR genes and the clinical implications of genetic variations in the prognosis interpretation remain unresolved. Consequently, further research is warranted to ascertain whether there are variations in the benefits derived from PARPi-targeted therapy among patients harboring different types of HRR mutations. We designed this prospective cohort study by using a retrospective and prospective research approach to collect information. In addition, in the PFS evaluation conducted within this study, patients undergoing maintenance therapy with PARPi may also receive other subsequent treatments, such as secondary debulking surgery, introducing potential confounding factors. Continuous monitoring of HRR mutation patterns throughout the disease trajectory is crucial for understanding resistance mechanisms and guiding future treatment strategies. Future research should focus on further validating the associations between HRR gene mutations and HRD status, as well as improving patient selection and personalized treatment approaches. Future research directions should also be emphasized on employing multiomics approaches or AI-based predictive modeling for HRD assessment.

## 7. Conclusion

This study investigates the relationship between HRR gene mutations and survival prognosis in OC patients, supplemented by essential clinical data. The identification of HRR gene mutations harbors potential as a prognostic marker for OC, enabling refined assessment techniques for HRD and pinpointing patients who are likely to exhibit a favorable response to PARPi therapy.

## Figures and Tables

**Table 1:** Baseline characteristics of all patients.

Characteristic	HRRmt (*N* = 70)	HRRwt (*N* = 28)	*p* value	BRCAwt (*N* = 63)	BRCAmt (*N* = 35)	*p* value
Age (years)	57.41 ± 12.497	57.29 ± 10.505	0.021	58.6	55.17	0.171
Stage						
I–II	9 (12.90%)	7 (25.00%)		13 (20.60%)	3 (8.60%)	
III	47 (67.10%)	16 (57.10%)	0.956	37 (58.70%)	26 (74.30%)	0.997
IV	14 (20.00%)	5 (17.90%)	0.852	13 (20.60%%)	6 (17.10%)	0.853
Histology type						
High-grade serous	59 (84.30%)	21 (75.00%)	0.360	47 (74.60%)	33 (94.20%)	0.151
Others	11 (15.70%)	7 (25.00%)		9 (14.20%)	0	
Largest primary tumor (cm)						
Unknown	10 (14.29%)	5 (17.85%)		1 (1.60%)	4 (11.40%)	
> 10	18 (25.71%)	4 (14.29%)	0.999	12 (19.00%)	12 (34.30%)	0.999
≤ 5	24 (34.29%)	11 (39.29%)	0.902	24 (38.10%)	11 (31.40%)	0.743
5–10	18 (25.71%)	8 (28.57%)	0.494	26 (41.30%)	8 (22.90%)	0.371
CA125-before surgery (U/mL)						
< 1000	49 (70.00%)	23 (82.10%)		49 (77.80%)	22 (62.90%)	
≥ 1000	21 (30.00%)	5 (17.90%)	0.376	14 (22.20%)	13 (37.10%)	0.327
CA125-after surgery (U/mL)						
< 105	30 (42.90%)	20 (71.40%)		36 (57.10%)	12 (34.30%)	
≥ 105	40 (57.10%)	8 (28.60%)	0.33	27 (42.90%)	23 (65.70%)	0.304
Residual lesions (cm)						
R0	36 (51.40%)	15 (53.60%)	0.520	33 (52.40%)	18 (51.40%)	0.44
R1–R2	24 (34.30%)	9 (32.10%)		30 (47.60%)	17 (48.60%)	
Pelvic LN metastasis						
Yes	36 (51.42%)	8 (28.57%)	0.204	24 (38.09%)	21 (60.00%)	0.237
No	34 (48.58%)	20 (71.43%)		39 (61.91%)	14 (40.00%)	
Involved lesion sides						
Bilateral	43 (61.40%)	16 (57.10%)		39 (61.90%)	20 (57.10%)	
Single	27 (38.60%)	12 (42.90%)	0.536	24 (38.10%)	15 (42.90%)	0.488
Metastasis to other organs						
Yes	14 (20.00%)	5 (17.90%)	0.788	30 (47.61%)	14 (40.00%)	0.747
No	56 (80.00%)	23 (82.10%)		33 (52.39%)	21 (60.00%)	
Chemotherapy regimens of NAC						
Cisplatin based	9 (12.86%)	3 (10.71%)		10 (15.90%)	3 (8.60%)	
Carboplatin based	60 (85.71%)	25 (89.29%)	1	53 (84.10%)	31 (88.60%)	1
Others	1 (1.43%)	0	1	0	1 (2.90%)	1
PARPi treatment						
Olaparib	15 (21.40%)	0		0	15 (42.90%)	
Niraparib	15 (21.40%)	6 (21.40%)	0.998	15 (23.80%)	6 (17.10%)	0.998
No	40 (57.20%)	22 (78.60%)	0.835	48 (76.20%)	14 (40.00%)	0.788
Bevacizumab						
No	51 (72.86%)	22 (78.57%)		46 (73.00%)	26 (74.30%)	
Yes	19 (27.14%)	6 (21.43%)	0.24	17 (27.00%)	9 (25.70%)	0.208

## Data Availability

The original contributions presented in the study are included within the article/supporting information. Further information is available from the corresponding author upon request.

## Supporting Information

Supporting table 1: Type of mutation and significance of mutations in recurrent patients of FIGO Stages III-IV.
