# Supplementary material for: The Clinical Pathological Characteristics and Prognostic Relevance of Homologous Recombination Repair Gene Mutations in Ovarian Cancer Patients: A Prospective Cohort Study
Source: Obstet Gynecol Int. 2025 Mar 24;2025:5578247. doi: 10.1155/ogi/5578247 (PMC11957853; doi:10.1155/ogi/5578247)
Supplement: Supporting Information — Additional supporting information can be found online in the Supporting Information section. [file 5578247.f1.docx]

| NO | mutant gene | position | cDNA changes | protein changes | mutation type | mutation significance |
| --- | --- | --- | --- | --- | --- | --- |
| 1 | BRCA1 | Exon 11 | c.2728del | p.Q910Kfs*90 | frameshift insertion | 5 |
| 2 | BRCA1 | Exon11 | c.3188C>A | p.S1063Y | missense mutations | 3 |
|  | BRCA2 | Exon11 | c.2683G>A | p.A895T | missense mutations | 3 |
| 3 | BRCA1 | Exon11 | c.3188C>A | p.S1063Y | missense mutations | 3 |
|  | BRCA2 | Exon11 | c.2683G>A | p.A895T | missense mutations | 3 |
| 4 | BRCA1 | Exon 11 | c.1961dup | p.Y655Vfs*18 | frameshift insertion | 5 |
| 5 | BRCA1 | Intron3 | c.135-2A>C | …... | Splice site mutation | 5 |
| 6 | BRCA1 | Exon7 | c.335del | p. N112Ifs*7 | frameshift insertion | 5 |
| 7 | FANCA | Exon41 | c.4087A>T | p.K1363* | nonsense mutation | 4 |
|  | FANCA | Exon19 | c.1772G>A | p.R591Q | missense mutation | 3 |
|  | RAD51D | Intron1 | c.82+5_82+13delinsT | …… | Splice site mutation | 3 |
| 8 | ERBB2 | Exon26 | c.3231C>T | p.P1077= | synonymous mutation | 3 |
| 9 | CDK12 | Exon4 | c.2206G>T | p.G736* | nonsense mutation | 4 |
|  | TP53 | Exon5 | c.493C>T | p.Q165* | nonsense mutation | 4 |
|  | ERBB2 | Exon12 | c.1320C>A | p.A440= | synonymous mutation | 3 |
|  | BRIP1 | Exon11 | c.1556T>C | p.I519T | missense mutation | 3 |
| 10 | CDK12 | Intron4 | c.2249-10G>A | …… | splice site mutation | 3 |
|  | BRIP1 | Exon10 | c.1352C>T | p.A451V | missense mutation | 3 |
| 11 | TP53 | Intron5 | c.559+1G>T | …… | missing insertion mutation | 4 |
|  | KRAS | Exon4 | c.327A>G | p.V109= | synonymous mutation | 3 |
| 12 | TP53 | Exon7 | c.767_768delinsTCATC | p.T256delinsII | missing insertion mutation | 3 |
|  | MRE11 | Exon5 | c.394C>T | p.(P132S) | missense mutation | 3 |
| 13 | BRIP1 | Exon3 | c.168dup | p.L57Tfs*12 | frameshift insertion | 4 |
|  | BRCA2 | Exon11 | c.4624G>T | p.V1542L | missense mutation | 3 |
| 14 | BRCA1 | Intron21 | c.5332+4dup | …… | splice site mutation | 3 |
|  | TP53 | Exon7 | c.772G>A | p.E258K | missense mutation | 4 |
|  | RAD54L | Exon19 | c.2063G>A | p.R688H | missense mutation | 3 |
| 15 | BRCA1 | Exon11 | c.2248_2252del | p.L750Vfs*10 | frameshift insertion | 5 |
|  | TSC2 | Intron4 | c.336+17C>T | …… | splice site mutation | 3 |
|  | MSH6 | Exon1 | c.169C>T | p.P57S | missense mutation | 3 |
|  | PALLD | Exon16 | c.2764G>C | p.V922L | missense mutation | 3 |
|  | TP53 | Exon5 | c.377A>G | p.Y126C | missense mutation | 3 |

Supplementary Table 1: Germline Pathogenic Variants Identified in Figo late-stage Patients Who recurred
